# Deciphering the Mutilation: Skillful Orthodontic Treatment Using Temporary Anchorage Devices and Asymmetric Extractions for a Patient With a Knife-Edge Ridge

**DOI:** 10.7759/cureus.67016

**Published:** 2024-08-16

**Authors:** Hitesh Ramdas Sawant, Parag Vishnu Gangurde, Shashank Sharad Gaikwad, Harsh Ashok Mishra, Vaishnavi Ravindra Kasarpatil, Diksha Vinod Wali, Shreya Kiran Koleshwar

**Affiliations:** 1 Department of Orthodontics and Dentofacial Orthopedics, Bharati Vidyapeeth (Deemed to be University) Dental College and Hospital, Navi Mumbai, IND

**Keywords:** temporary anchorage devices (tads), orthodontic, mutilated dentition, implants, knife-edged ridge

## Abstract

One of the biggest challenges in orthodontic management of adult patients is the presence of mutilated dentition, which is characterized by severe tooth loss and subsequent tipping of adjacent teeth or extruding of antagonists, worsening the malocclusion. As they offer a solid anchor for intricate tooth movement, temporary anchorage devices (TADs) have proven invaluable to orthodontic treatment. In this case study, we highlight the importance of TADs in getting the best possible treatment outcomes while describing the effective management of a patient with mutilated dentition. TADs made it possible to control tooth movement precisely, restoring esthetics and functional occlusion. This case report emphasizes the value of TADs in contemporary orthodontic practice and shows their potential for correcting mutilated dentition.

## Introduction

Orthodontic treatment for a mutilated dentition is challenging because of various factors like missing teeth, supraerupted teeth of the opposing arch, carious teeth, or teeth with prostheses. Long-standing edentulous areas are characterized by knife ridges in which prosthetic rehabilitation with implants is difficult [1]. The split ridge method combined with bone graft augmentation is necessary in these cases to establish a suitable recipient site for implant insertion [2]. In patients reluctant to undergo invasive procedures like the ridge split technique and in whom orthodontic treatment is indicated, such spaces can be closed by orthodontics with the help of temporary anchoring devices (TADs) [3,4]. Given the complications associated with various invasive procedures, a treatment plan that best meets the patient's needs with the least amount of invasion is the priority established by both the patient and the operator, particularly in cases of mutilated dentition.

TADs can effectively increase the anchorage without placing undue strain on the anchor unit [5]. This case study demonstrates how to treat a patient whose dentition has been severely mutilated by employing TADs as a key component of the treatment plan and avoiding the necessity for invasive prosthetic operations. The aforementioned case is being handled with consideration for both esthetics and function by asymmetric extraction [6] and careful use of anchorage. Orthodontic tooth movement, particularly in the knife-edge ridge region, has aided in periodontal remodeling [7]. Our goal is to demonstrate, via a thorough account of the case and its resolutions, the important role that TADs play in obtaining favorable outcomes in a mutilated orthodontic case [7].

## Case presentation

**Figure 1:**
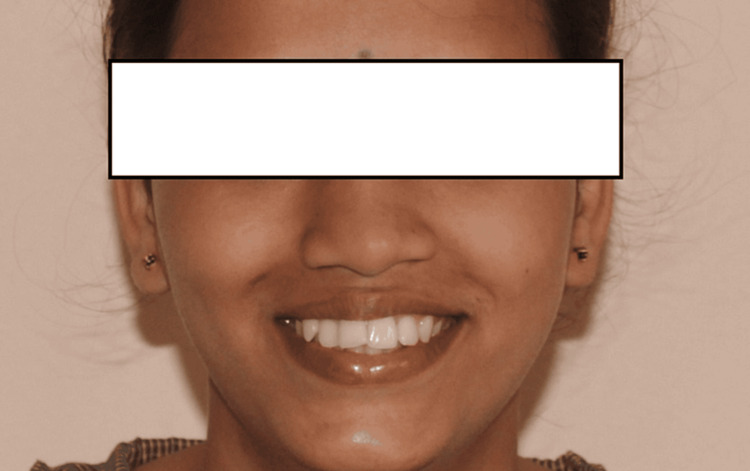
Preoperative profile picture

**Figure 2:**
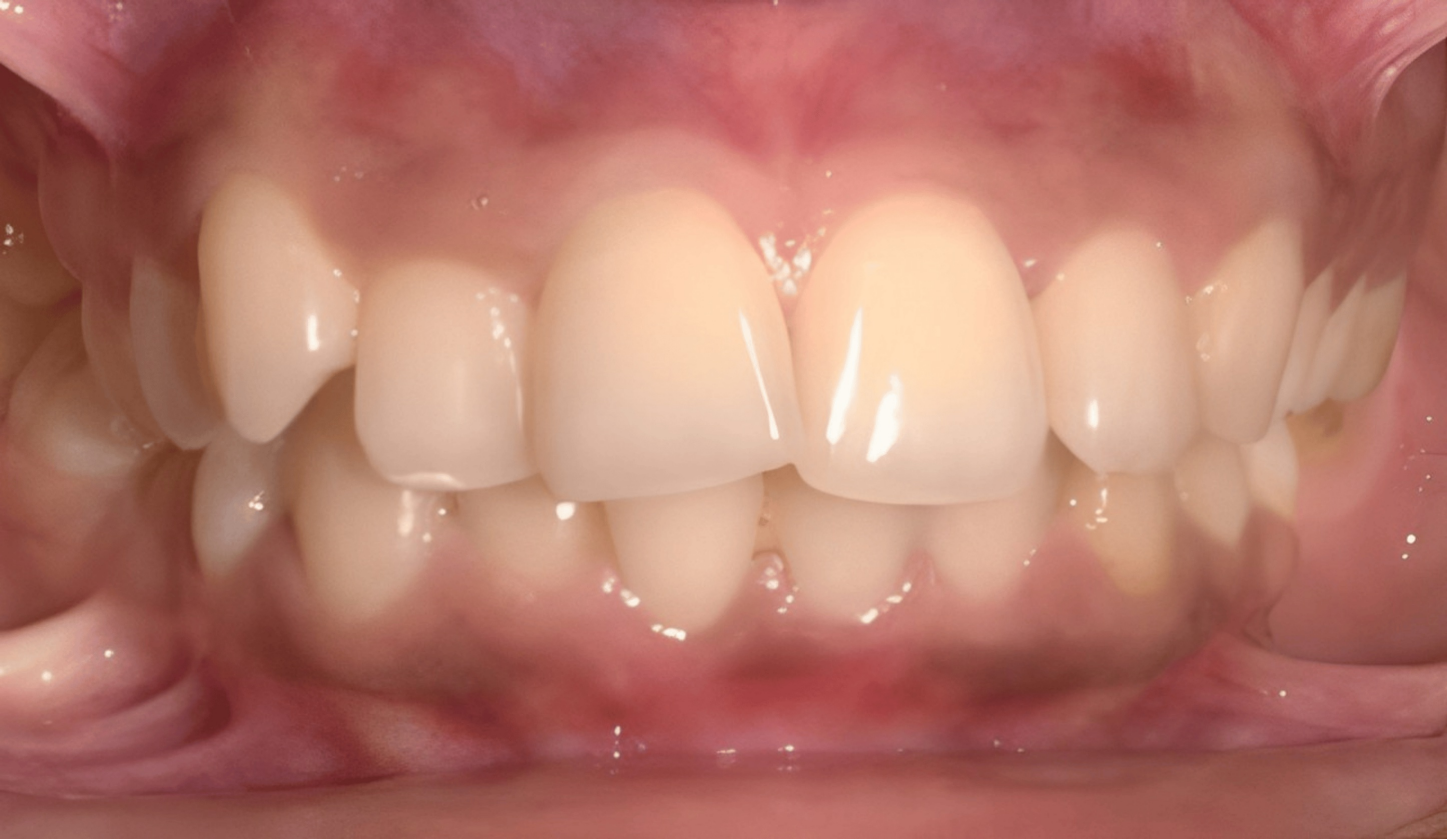
Intraoral image of frontal view

**Figure 3:**
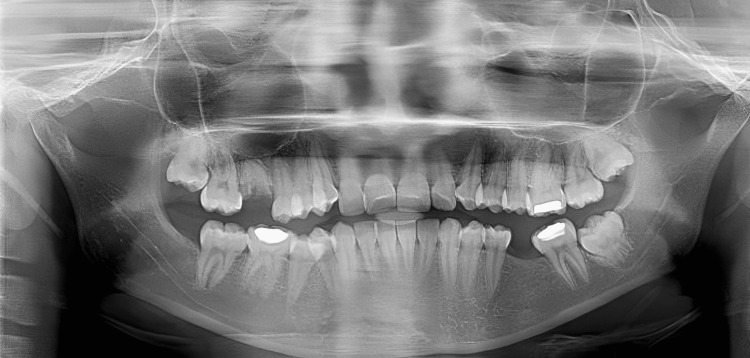
Preoperative OPG of the patient OPG: orthopantomography

**Figure 4:**
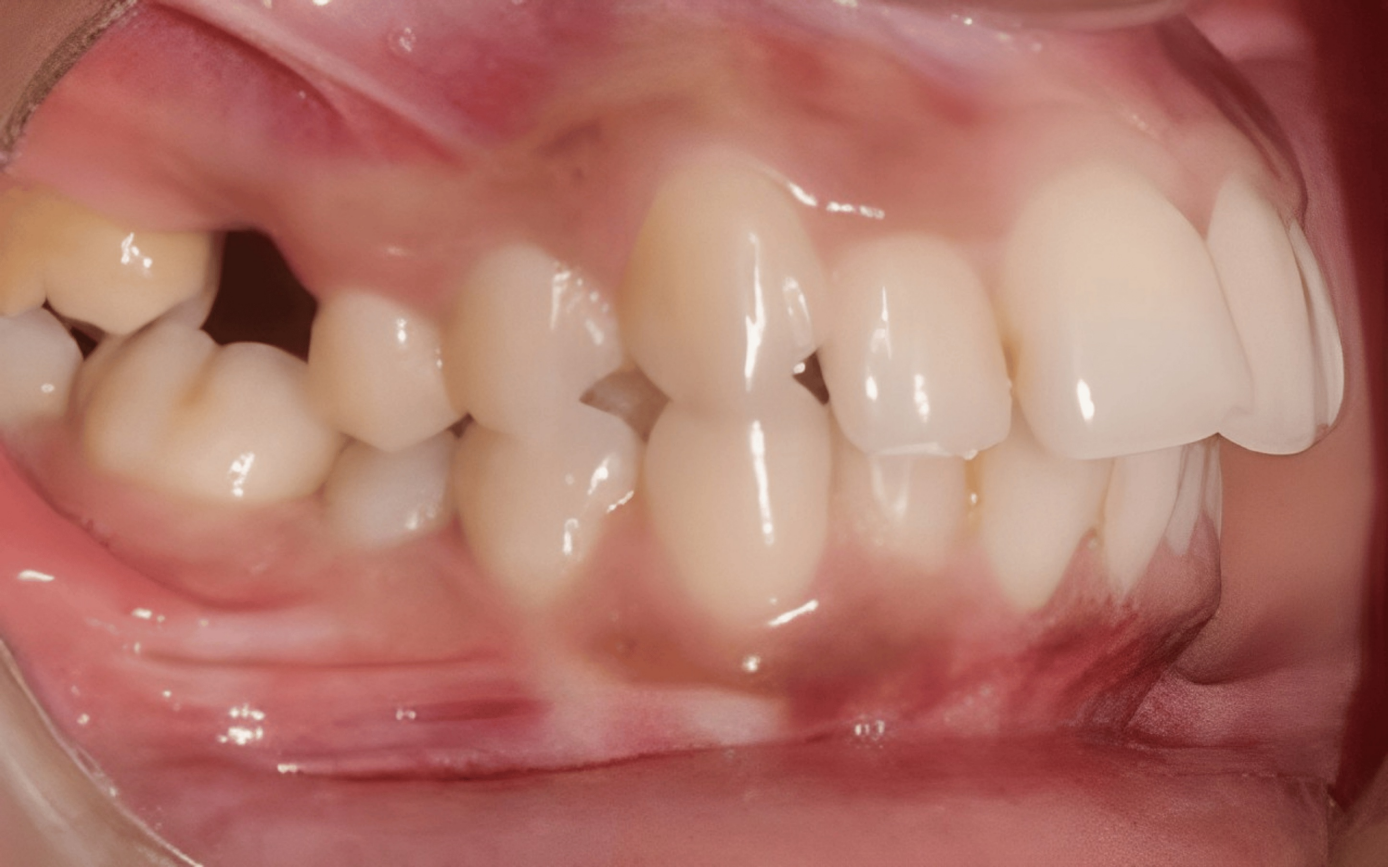
Intraoral preoperative lateral view

The mutilated dentition (root piece 16, missing 36, and extruded 14 and 15 segments) is corrected using TADs in this case report. The primary complaint of a 25-year-old female patient who presented to the Orthodontics and Dentofacial Orthopedics Department was that her front teeth were positioned forward and irregularly. She was also concerned about the spaces in her 16 and 36 regions. The patient's skeletal class II maxillomandibular relationship included an orthognathic maxilla and retrognathic mandible, decreased lower third facial height, average maxillary posterior height, and average growth pattern. Proclined and protruded maxillary and mandibular incisors and a knife-edge ridge are present in the 16 and 36 regions. Additionally, there was crowding in the maxillary dentition (3.5 mm) and mandibular dentition (2 mm) with extraction spaces of 7.5 mm in the 36 region and 8 mm in the 6 region, overjet (3 mm) and overbite (3 mm), extruded 14 and 15, acute nasolabial angle, average mento labial sulcus, and potentially competent lips (Figures 1-4).

The treatment objectives for the patient included correcting the proclined and protruded upper and lower incisors (taking cognizance of pretreatment cephalometric values for dental proclination, as enlisted in Table 1), managing the knife-edge ridge, achieving a class I canine relationship bilaterally after treatment (pretreatment intraoral photos reveal the presence of an end-on canine relationship on the right side and a class I canine relationship on the left side in accordance with Figure 2, which needs to be corrected by orthodontic intervention), relieving crowding in the upper and the lower arch and managing the extruded 14 and 15 while uprighting 37. Special considerations for managing the knife-edge ridge included comprehensive radiographic and clinical evaluations to assess ridge morphology, followed by potential bone grafting to augment deficient areas. Orthodontic mechanics involved the application of light and controlled forces to avoid compromising the ridge's integrity, while meticulous periodontal care ensured healthy soft-tissue coverage. Collaboration with periodontists and oral surgeons facilitated a holistic treatment plan customized to the patient’s unique anatomical needs, ensuring stability and health of the ridge throughout the orthodontic process. The treatment plan involved extracting teeth 24 and 45, aligning and leveling, uprighting of 37, and retracting the maxillary and mandibular anterior teeth together. An alternative treatment option considered extraction of teeth 14, 24, 34, and 45, followed by prosthetic replacement of teeth 16 and 36. This treatment option was not executed as the patient had already been subjected to the brunt of extractions in relation to 16 and 36 before starting orthodontic treatment. Hence, it is prudent to devise orthodontic treatment mechanics that would utilize extraction spaces 16 and 36 for orthodontic alleviation of crowding and proclination. Also, this satisfies the psychological instinct of the patient to prevent extractions in quadrants subjected previously to extraction. Also, the need for prosthetic replacement of 16 and 36 by implants gets eliminated by the selected treatment plan, thus reducing the effective treatment time as well as the cost of the overall treatment.

**Figure 5:**
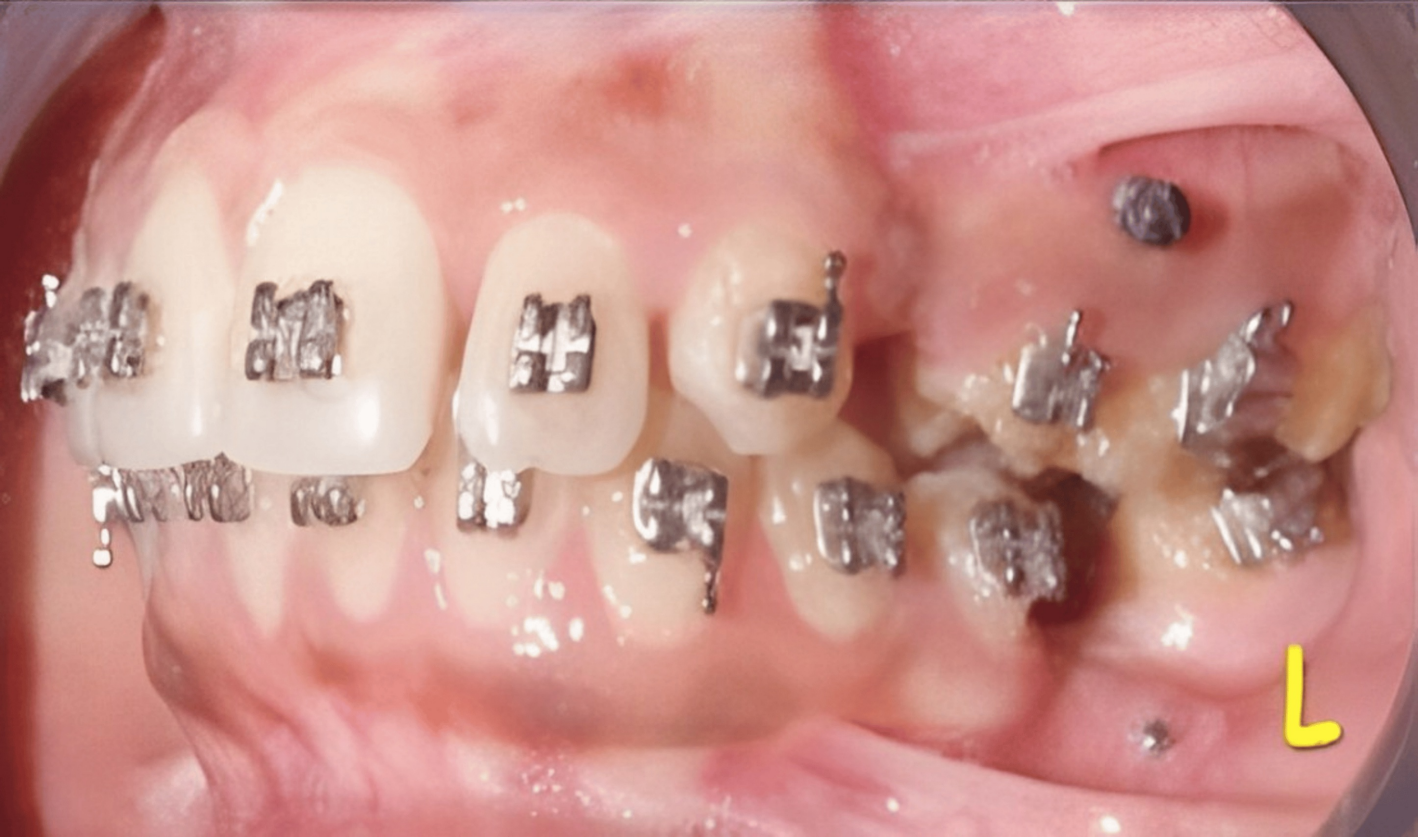
TAD placement in the left lateral view L: left side; TAD: temporary anchorage device

Anchorage considerations were critical in both the right and left sides of the maxillary arch and the left side of the mandibular arch, which was augmented by using TADs. Management of extruded 14 and 15 was crucial in relation to establishing proper occlusion. Bracket positioning in relation to 14 and 15 was done meticulously to prevent inadvertent extrusion. Care was taken to prevent gingival positioning of brackets as these would lead to extrusion of dentoalveolar segments (Figure 5).

Before initiating the active orthodontic therapy, the patient provided written informed consent. Bonding was performed using 0.022” × 0.028” slots in the McLaughlin, Bennett, and Trevisi prescription. This approach aimed to address the patient's specific orthodontic concerns effectively. The treatment plan was carefully devised to achieve the desired dental corrections while considering the patient's oral health and functional requirements. Regular monitoring of treatment progress and adjustments were integral parts of the orthodontic process to ensure optimal outcomes and patient satisfaction. Careful assessment of knife-edge ridge in relation to 16 and 36 was done by clinical evaluation in a sequential manner. The use of optimum orthodontic force throughout the treatment reduces hyalinized zones, which are responsible for delayed orthodontic tooth movement. Also, careful execution of planned treatment mechanics helped eliminate the need for splitting of the alveolar ridge and placement of the bone graft.

Extractions were performed asymmetrically, involving tooth 16 in root pieces, along with teeth 24 and 45, all under local anesthesia before bonding the fixed orthodontic appliance. Subsequently, alignment and leveling procedures were performed in the maxillary and mandibular arches. Anchorage proved critical, particularly in the upper arch on both sides and the lower left side. It was noted that following normal retraction mechanics could result in undue stress on tooth 16 in the first quadrant. To address this concern and accommodate space requirements in the upper arch after extraction, TADs were strategically employed to enhance anchorage, utilizing a force magnitude of 150 g.

Additionally, mesially tipped tooth 37 was deemed unsuitable for direct use as anchorage for the retraction of the lower left segment. Consequently, anchorage in the lower left segment was reinforced with TADs under infiltration anesthesia using 2% lignox (lignocaine with adrenaline). TADs of 1.4 × 6 mm were positioned interdentally between 25 and 26, and mesial to 17 and 47, respectively. The successful en masse retraction of the maxillary and mandibular anterior teeth was successfully achieved through the aforementioned treatment mechanics.

Treatment result

**Figure 6:**
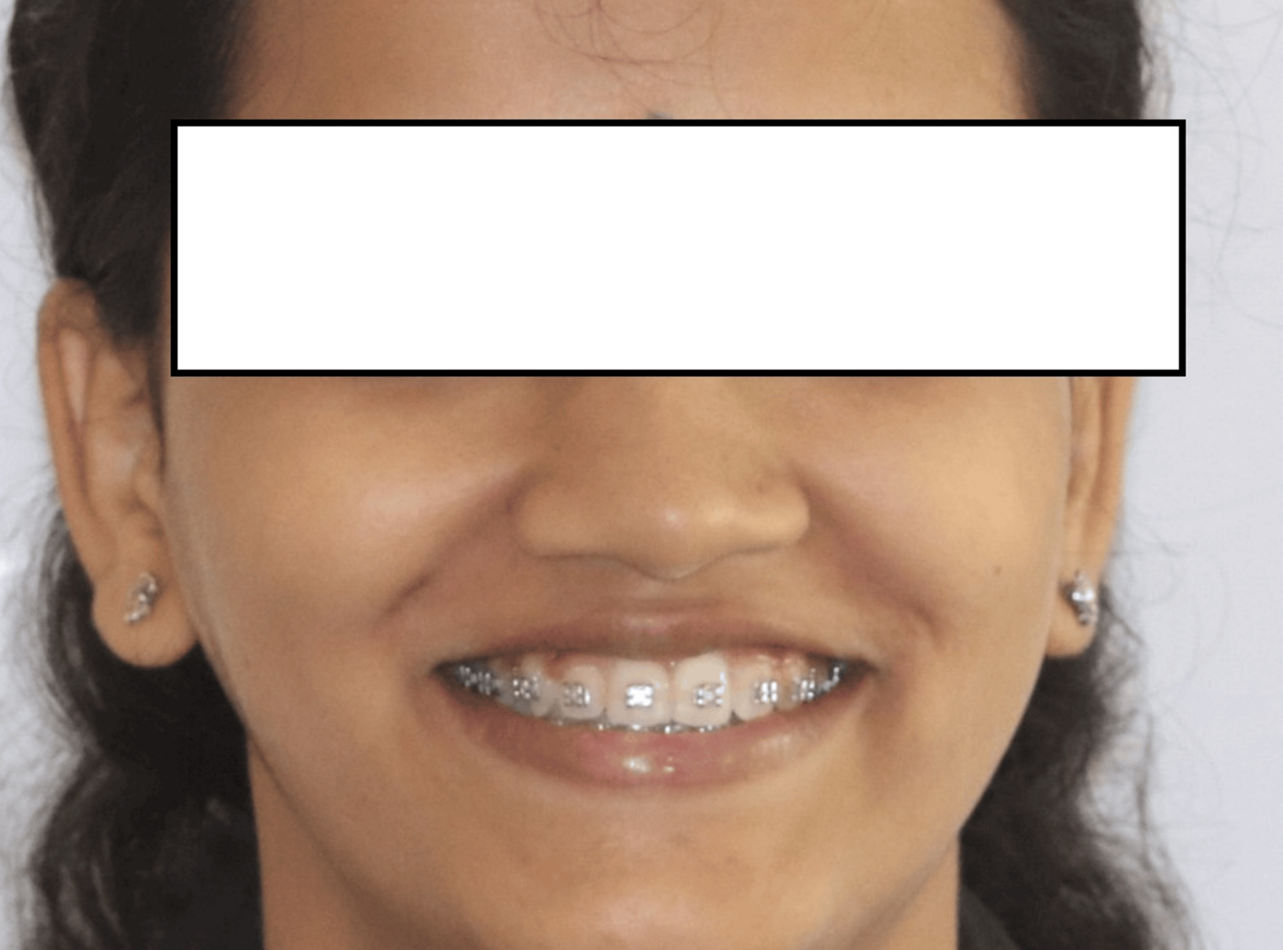
Intraoperative smile

**Figure 7:**
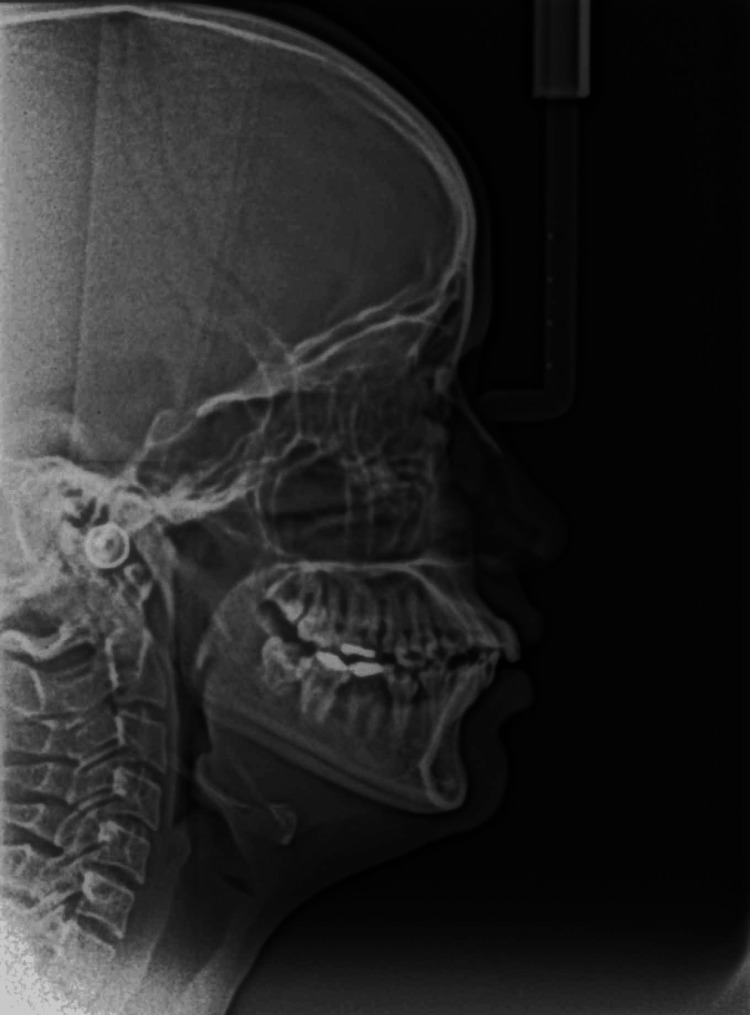
Preoperative lateral cephalography

**Figure 8:**
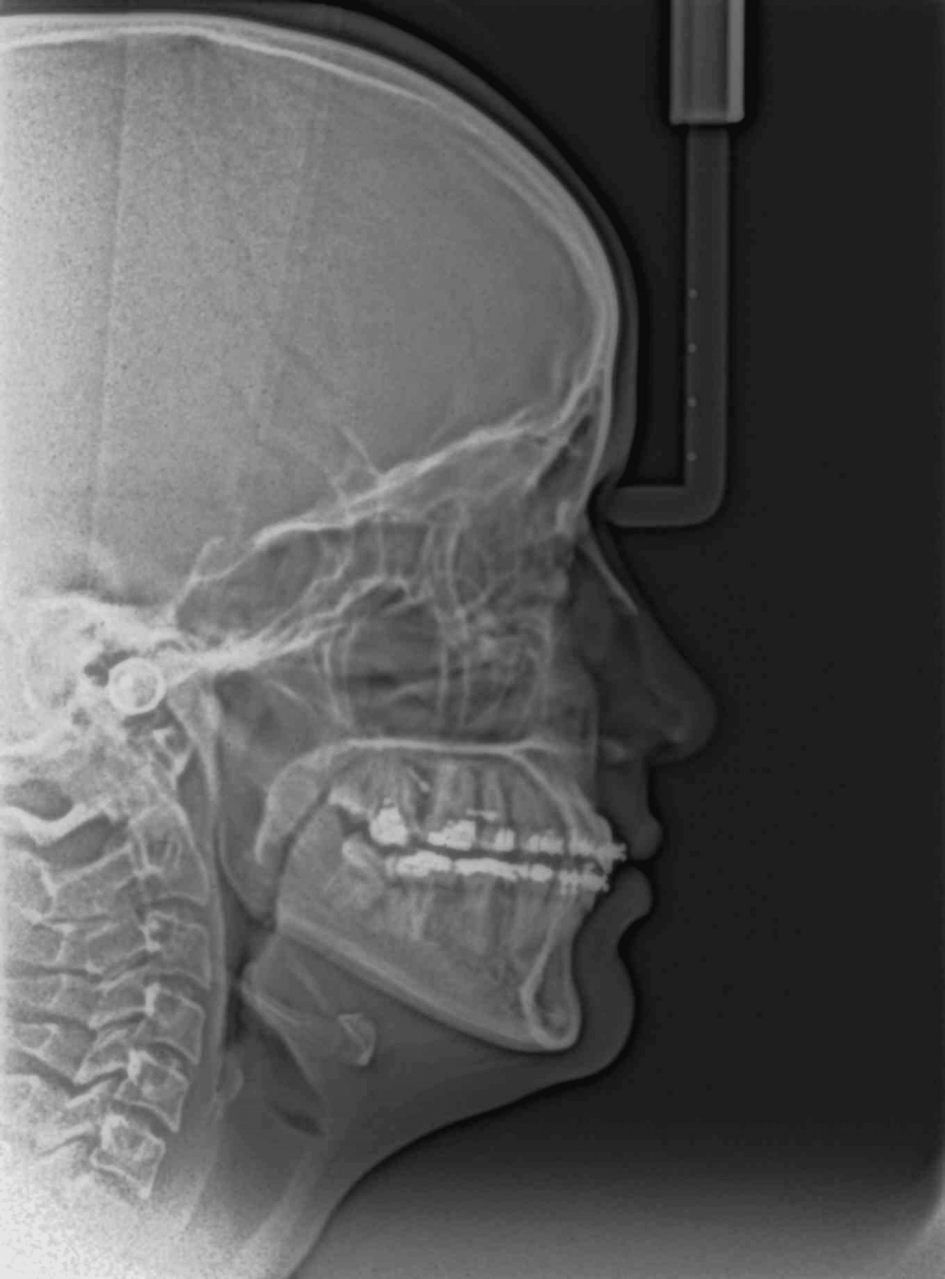
Postoperative lateral cephalography

**Figure 9:**
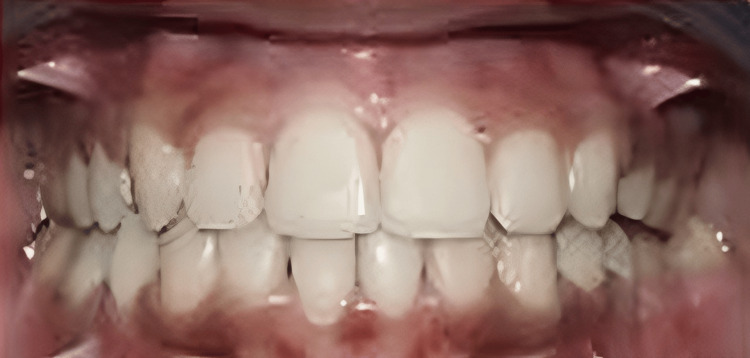
Postoperative intraoral frontal view

**Figure 10:**
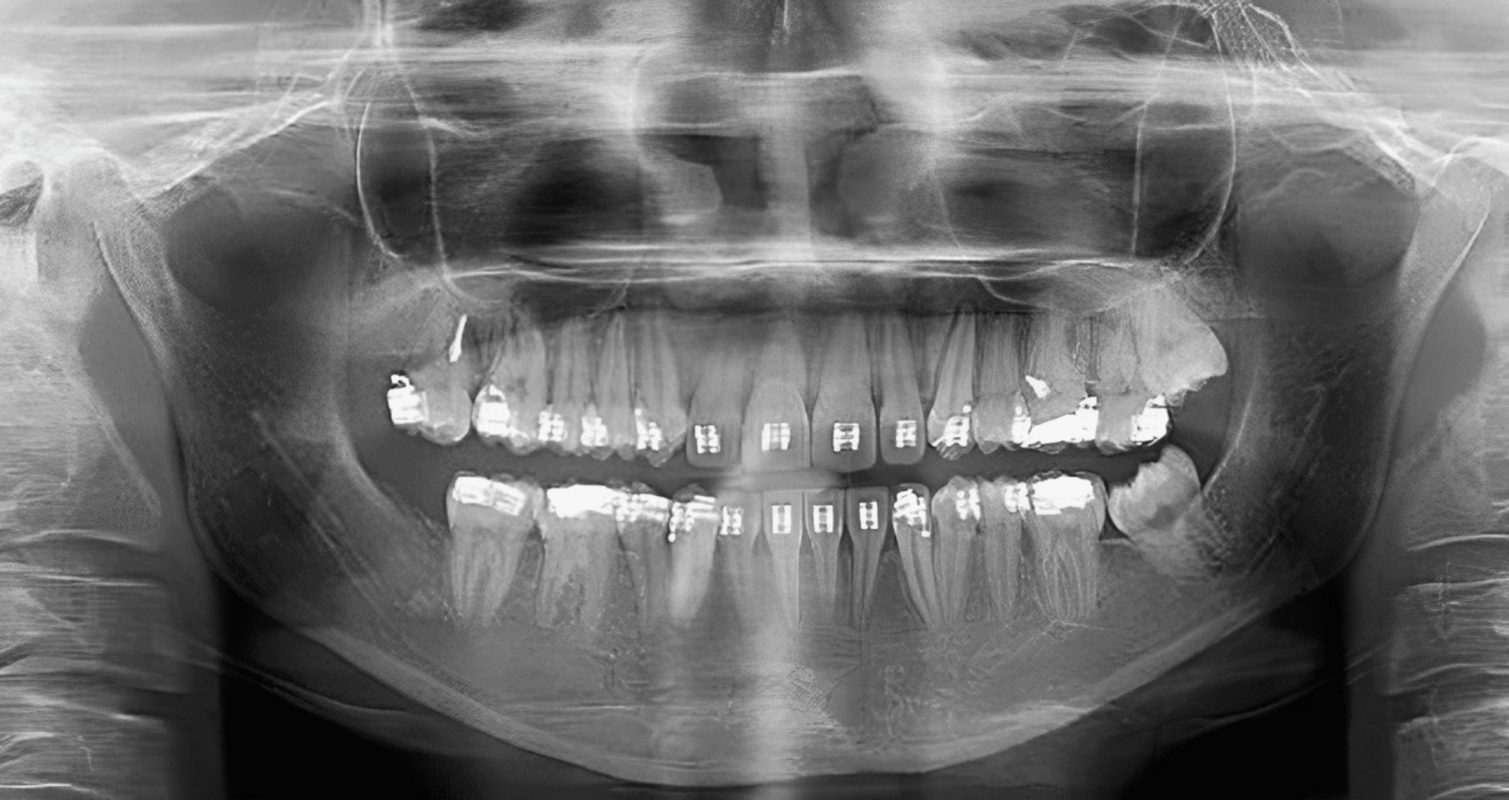
Postoperative OPG of the patient OPG: orthopantomography

At the end of the treatment, the occlusion was settled with class I canine relation bilaterally with 2 mm of overjet and overbite, and the changes were also observed in the lateral cephalogram (Figures 6-10).

There was a midline discrepancy seen of the lower midline shifted toward the right side by 2 mm. However, this can be attributed to the fact of asymmetric extractions carried in the upper and the lower arch (Table 1).

**Table 1:** Cephalometric values during the treatment duration SNA angle: sella-nasion-A point angle; SNB angle: sella-nasion-B point angle; ANB angle: A point-nasion-B point angle; wits appraisal (mm): a cephalometric measurement indicating an anteroposterior jaw relationship; N perp to point A (mm): perpendicular distance from nasion to point A; N perp to pog (mm): perpendicular distance from nasion to pogonion; Go-Gn to SN: gonion-gnathion to sella-nasion; UI to NA angle: upper incisor to NA angle; UI to NA (mm): upper incisor to NA distance; UI to SN angle: upper incisor to SN angle; LI to NB angle: lower incisor to NB angle; LI to NB (mm): lower incisor to NB distance; LI to A pog (mm): lower incisor to A-pogonion distance; LI to Mand. plane angle: lower incisor to mandibular plane angle

Variable	Normal values	Pretreatment	Posttreatment
Skeletal relationship			
SNA angle	82°	81°	79°
SNB angle	80°	77°	76°
ANB angle	20	40	30
Wits appraisal	+1 mm	+4.8 mm	+3.7 mm
N perp to point A	0 + 2 mm	-1 mm	0.9 mm
N perp to pog	0 to -4 mm	-6.8 mm	-4.7 mm
Go-Gn to SN	32°	36°	34°
Angle of inclination	85°	90°	87°
Lower anterior face height	67.2 + 4.7 mm	51.4 mm	54.9 mm
Effective maxillary length	93.6 + 3.2 mm	72.9 mm	80 mm
Effective mandibular length	121 + 4.5 mm	91.3 mm	98 mm
y-axis angle	66°	66°	64°
Facial axis angle	90 + 3°	93°	95°
Sum of posterior angles	396 + 6°	396°	393°
Dental relationship			
UI to NA angle	22°	37°	30°
UI to NA	4 mm	7.8 mm	3.4 mm
UI to SN angle	102°	120°	105°
LI to NB angle	25°	34°	30°
LI to NB	4 mm	8 mm	4.5 mm
LI to A pog	1-2 mm	5.3 mm	2.2 mm
LI to Mand. plane angle	90°	99°	96°
Inter incisal angle	135°	105°	106°
Overjet	2 mm	3 mm	2 mm
Overbite	2 mm	3 mm	2 mm
Soft tissues			
S line to upper lip	0 + 2 mm	2.6 mm	0.6 mm
S line to lower lip	0 + 2 mm	1.1 mm	+1 mm
Nasolabial angle	90-110°	69°	90°

## Discussion

Adult seeking orthodontic treatment requires completion of their treatment in a time-bound manner. Failure to achieve this results in patient burnout. It is prudent to incorporate the use of TADs in such situations to reduce the need for compliance on the patient’s side and cause minimal taxing of anchorage of dental units.

This case report aimed to describe the correction of a dentition that was severely malformed, which presented several clinical challenges such as missing molars, adjacent posteriors that were mesially tipped (37), antagonists that were extruded (14 and 15), and a knife-edge ridge resulting from a 4-year long period following extraction. The patient, in this case, had a malocclusion that affected both function and appearance, and there was considerable bone loss in the 16 and 36 regions. The treatment strategy included asymmetric extraction of teeth 24 and 45, periodontal repair of the knife-edge ridge, and the prudent use of TADs for orthodontic tooth movement to establish a harmonic occlusion and an esthetically acceptable smile [8].

Because they offered crucial anchorage, TADs proved invaluable in this situation. Teeth might be moved carefully with TADs without affecting the placements of neighboring teeth. This made it possible for the orthodontist to rectify rotations, close spaces, and intrude on certain teeth when necessary. The achievement of the intended therapeutic outcomes was greatly aided by the capacity to perform such exact and detailed motions. The success of TADs depends on various factors like the skill of the operator in the placement of TADs, patients' compliance with maintenance of oral hygiene, and the need for replacement in case of failure of TADs.

Additionally, the use of TADs lessened the requirement for patient compliance because standard orthodontic mechanics, such as the lingual arch or Nance button, can occasionally be cumbersome for the patient and cause patient fatigue in the early stages of treatment [9]. TADs eliminate the need for patient cooperation, which allows the treatment plan to be completed on schedule [10]. The orthodontic team reduced the amount of anchorage on molars by using TADs, giving them more control over the treatment process. The interdisciplinary method for managing the knife-edge alveolar ridge with invasive operations like ridge split followed by implant placement or traditional fixed partial denture was abandoned in favor of orthodontic treatment with TADs [11,12]. The effective management of the knife-edge ridge in the aforementioned case can be considered a viable option for effectively modifying the width of alveolar bone with dimensional alterations in edentulous ridges.

## Conclusions

This case report concludes by highlighting the important part TADs play in restoring severely mutilated dentition. In this case, we successfully corrected the proclined and protruded upper and lower incisors, managed the knife-edge ridge, achieved a class I canine relationship bilaterally after treatment, relieved crowding in the upper and lower arch, and also managed the extruded 14 and 15 while uprighting 37. This was all made possible through the exact control and stability provided by TADs, leading to the successful restoration of functional occlusion and esthetics in this complicated instance.
